# Torsion of an Encysted Fluid Collection

**DOI:** 10.1100/tsw.2007.152

**Published:** 2007-04-09

**Authors:** Atilla Senayli, Yesim Senayli, Engin Sezer, Taner Sezer

**Affiliations:** ^1^ Department of Pediatric Surgery, University of Gaziosmanpasa, Araştırma ve Uygulama Hastanesi, 60080, Tokat, Turkey; ^2^ Department of Anesthesiology and Reanimation, University of Gaziosmanpasa, Araştırma ve Uygulama Hastanesi, 60080, Tokat, Turkey; ^3^ Department of Dermatology, University of Gaziosmanpasa, Araştırma ve Uygulama Hastanesi, 60080, Tokat, Turkey; ^4^ Department of Pediatrics, University of Gaziosmanpasa, Araştırma ve Uygulama Hastanesi, 60080, Tokat, Turkey

**Keywords:** torsion, hydrocele, acute scrotum, color Doppler sonography

## Abstract

Torsion of a cyst within the tunica vaginalis is a rare entity and clinical course can easily be confused with other diseases that cause acute scrotum. We report a 6-year-old child with 3 days of acute scrotum findings. Patient had surgery with the suspicion of testis torsion. Torsion of a cyst within the tunica vaginalis was found intraoperatively. In pathologic evaluation, a necrotic funicular cyst was diagnosed. Two different mechanisms were reported for the reason of this disease: hernia sac protrusion in the hydrocele sac and bell-clapper deformity. Our observations were on the side of bell-clapper deformity. We aimed to share our findings with this report.

Torsion of a cyst within the tunica vaginalis is a new and very rare entity represented by only eight cases in the medical literature[1]. The mechanism underlying the torsion is unknown, but two possible explanations have been reported: (1) a bell-clapper deformity and (2) protrusion of a hernia sac in the hydrocele[2,3]. The correct diagnosis is often difficult based on acute scrotal findings[4]. Consequently, all of the cases involving torsion of a cyst that have been reported in the literature are based on operative findings.

We describe herein a case involving a 6-year-old child diagnosed with torsion of a cyst within the tunica vaginalis. The purpose of this communication was to share our intraoperative observations regarding the pathogenesis of the condition and compare our observations with those in the literature. We noted some differences from the cases reported in the literature as it seemed that our patient had a cyst in the tunica vaginalis and we suggest that our observations were consistent with a bell-clapper deformity.

## CASE REPORT

A 6-year-old boy was admitted to our hospital with a 3-day history of right-sided scrotal pain, tenderness, and swelling. There was no history of scrotal trauma, medical or surgical treatments, or related family diseases. He had no history of high fever, nausea, or vomiting. On physical examination, we found an enlarged, tender, right hemi-scrotum without fluctuation. The left hemi-scrotum and inguinal region were normal. There was no abdominal tenderness or guarding. No radiodiagnostic testing was performed.

Based on the history and physical examination, testicular torsion was the working diagnosis and an urgent, right inguinal exploration was performed. Following the spermatic cord structures, a tender and edematous tunica vaginalis was identified and opened, from which a small amount of serous fluid was drained. An approximately 4- × 3- × 1-cm necrotic and pedunculated cystic tissue was observed (Fig. 1). The cystic tissue was twisted at an angle of 540°, and found to be 5 mm long beyond the twisted peduncle of the cyst. No membranous structures were found along the spermatic cord and testis, which would have defined a hernia or hydrocele sac. We explored the internal inguinal ring and found no hernia sac at this level. The testis was normal except for moderate inflammation. A cystectomy was performed. Pathologic evaluation of the specimen revealed a necrotic funicular cyst. Scrotal swelling was minimal 2 weeks after the surgical procedure and there was no pain or other complaints by the patient at that time.

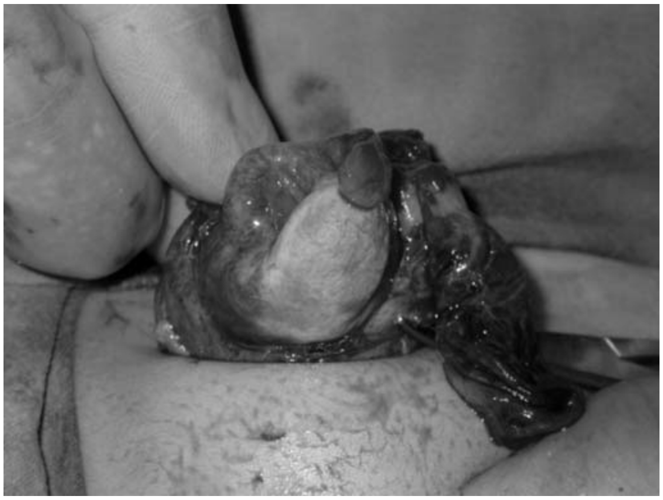

**FIGURE 1.** Intraoperative photograph during inguinal exploration with necrotic cyst. There was no membranous tissue representing the wall of hernia or hydrocele sac on the testis or testicular structures.

## DISCUSSION

Acute scrotal pain is associated with testicular torsion, torsion of an appendage, and epididymoorchitis in 39.5, 35–67, and 15.5% of patients, respectively[1,5]. Differential diagnoses for acute scrotal pain include conditions such as torsion of the spermatic cord, incarcerated inguinal hernia, torsion of the appendix of the testis, torsion of the appendix of the epididymis, acute testitis, acute epididymitis, lymphangioma, or torsion of a benign cyst from the tunica vaginalis testis[2]. Variable clinical presentations, however, can make the precise diagnosis of acute scrotal pain difficult, often necessitating surgical exploration[2].

In the past several decades, a new entity, so-called torsion of a hernia sac, has been added to the anatomical variants encountered by the inguinal surgeon[4].Torsion of a hernia sac is a rare condition[1,4]; only eight cases having been previously reported[4]. Torsion of a hernia sac has been reported in boys 2–10 years of age, with a mean age at presentation of 5.7 years, including the patient described herein. Seventy-five percent of cases of torsion of a hernia sac are located on the right side, also as was the case for our patient[1].

In clinical practice, it is very difficult to distinguish torsion of a hernia sac from the afore-mentioned acute scrotal conditions[2]. One of the clinical differences is absence of gastrointestinal symptoms, which often occurs with spermatic cord torsions[3]. Contralateral scrotal swelling is another distinctive feature that occurs with torsion of a hernia sac, although we did not observe this in our patient[3]. Radiologic methods may facilitate an accurate diagnosis of acute scrotal pain; indeed, some authors consider color Doppler sonography a significant improvement in the assessment of testicular torsion[6]. In one study, a spiral twist of the cord at the external inguinal ring was used to establish the diagnosis of testicular torsion, thereby avoiding an unnecessary surgical procedure[7]. Because definitive ultrasonographic characteristics of torsions involving cysts or hydroceles have not been defined, surgical exploration continues to have a much higher sensitivity than ultrasound[1]. From another point of view, missing a diagnosis of torsion remains an important legal problem in pediatric surgery departments[6]. As a result, all authors agree that surgical exploration is mandatory when there is a suspicion of torsion[6].

The etiology of a cystic torsion within the tunica vaginalis is unclear. It is reported that a thin, membranous hernia sac may invaginate within a hydrocele, and then subsequently twist[2]. By such a mechanism, the base of the vaginal process is first closed by a membrane. Then, the closed membrane of the vaginal process protrudes into the hydrocele within the scrotum. Finally, torsion occurs suddenly due to an unknown cause[2]. During our surgery, we did not observe a membranous sac presenting as the inner wall of a hydrocele along the testis extending through the internal ring. We also did not find a vaginal process at the internal ring. In fact, the area of necrosis was detected after dissection of the tunica vaginalis, suggesting an encysted fluid collection in the tunica vaginalis testis.

Another possible mechanism to explain a cystic torsion within the tunica vaginalis may be the same as in the mechanism leading to torsion of a spermatic cord[3]. According to this theory, a communicating hydrocele is twisted with a bell-clapper deformity, in which the spermatic fascia completely encircles the distal spermatic cord and the hydrocele[3]. Accordingly, connecting tissues around the hydrocele and testis remain loose, causing free swinging and rotation of the hydrocele within the thickened internal spermatic fascia[3]. Our observations during surgery were consistent with this theory. We easily dissected the necrotic cyst from the circulating connective tissue. The cyst was detected inside the fascia surrounding the testicular structures. Nevertheless, there has only been one patient so described in the literature and that patient had a communicating hydrocele[3]. Although, this was the main clinical difference between our patient and that, a bell-clapper theory was nevertheless favored.

Making an accurate diagnosis of torsion of the testis, and in a timely fashion, is important because gonadal salvage is possible only if intervention occurs within 6 h of the onset of pain[5]. If torsion of the testis exists, urgent surgical management is indicated. If the diagnostic evaluation reveals a condition other than torsion, the patient may be managed expectantly. Although torsion of a cyst occurs infrequently in patients with an acute scrotum, if diagnosed preoperatively by taking a detailed history combined with radiologic findings, emergent surgery may be substituted with pain management.
